# Supplementary material for: Occurrence of Candidemia in Patients with COVID-19 Admitted to Five ICUs in France
Source: J Fungi (Basel). 2022 Jun 28;8(7):678. doi: 10.3390/jof8070678 (PMC9320616; doi:10.3390/jof8070678)
Supplement: Supplementary file 1 [file jof-08-00678-s001.zip › jof-1738957-supplementary.pdf]

Table S1. Characteristics of the study population

	Characteristics		All patients (n = 264)
**Demographic characteristics and underlying conditions**	Age, median (IQR), year		56 (48-64)
	Male Gender, n (%)		186/264 (70.5)
	Body Mass Index > 25 kg/m², n (%)		180/250 (72)
	Hypertension, n (%)		138/257 (53.7)
	Diabetes, n (%)		88/259 (34)
	Dyslipidemia, n (%)		50/258 (19.4)
	Active smoker, n (%)		18/249 (7.2)
**Preexisting risk factors for invasive fungal infection**	Hemopathy, n (%)		8/257 (3.1)
	Hematopoietic stem cell allografft, n (%)		3/257 (1.2)
	Solid organ transplant, n (%)		18/257 (7)
	Corticosteroid therapy >0.3 mg/kg, n (%)		8/257 (3.1)
	HIV infection, n (%)		8/257 (3.1)
**Specific COVID-19 therapy**	HCQ, n (%)	HCQ	61/245 (24.9)
		HCQ/AZT	10/245 (4)
	Antiviral, n (%)	Lopinavir/ritonavir	30/245 (12.2)
		Remdesivir	18/245 (7.3)
		Oseltamivir	17/238 (7.1)
	Anti-IL6, n (%)	Tocilizumab	13/242 (5.4)
		Sarilumab	3/245 (1.2)
	Anti-IL1, n (%)		1/245 (0.4)
**Corticosteroid therapy related to COVID-19 and/or ICU stay**	Before ICU admission, n (%)		41/234 (17.5)
	During ICU stay, n (%)		107/232 (46.1)
**ICU management and clinical characteristics, median (interquartile)**	ICU stay (n=221), median (IQR), days		30 (19-51)
	SAPS II score (n=196), median (IQR)		55 (39-68)
	Intubation period (n=200), median (IQR), days		27 (15-45)
	Worst P/F (n = 251), median (IQR)		60 (51-80)
	Extracorporeal membrane oxygenation, n (%)		153/244 (62.7)
	Vasopressor support (>0,5mg/h of noradrenalin), n (%)		172/233 (73.8)
	Dialysis, n (%)		75/234 (32)
**Inflammatory markers**	C-reactive protein (n=197), median (IQR), mg/L		171 (70-289)
	Ferritine (n=191), median (IQR), mg/L		1753 (831-3271)
	IL-6 (n=66), median (IQR), pg/mL		146 (44-564)
**Blood cultures**	Total numbers of blood cultures sampled per patient, median [min ; max]		12 [0 ; 132]
	Positive blood cultures per patient, median [min ; max]		1 [0 ; 30]
**Overall mortality, n (%)**			105/244 (43)

Definition of abbreviations: IQR = interquartile range; HCQ= hydroxychloroquine; AZT = azithromycine; ICU= intensive care unit; SAPS = Simplified Acute Physiology Score; P/F = PaO_2/FiO_2
